# C-reactive protein to albumin ratio as a novel predictor of stroke-associated pneumonia: a retrospective cohort study revealing non-linear threshold effects

**DOI:** 10.3389/fneur.2026.1786925

**Published:** 2026-04-09

**Authors:** Tingting Duan, Changwei Zou, Ming Yang, Jiangxingzi Xu, Zhaoping Wu

**Affiliations:** 1 Department of Neurology, The Quzhou Affiliated Hospital of Wenzhou Medical University, Quzhou People's Hospital, Quzhou, China; 2 Department of Ophthalmology, The Quzhou Affiliated Hospital of Wenzhou Medical University, Quzhou People’s Hospital, Quzhou, China

**Keywords:** acute ischemic stroke, C-reactive protein toalbumin ratio, generalized additive model, inflammatory biomarkers, nutritional status, risk stratification, stroke-associated pneumonia, threshold effect

## Abstract

**Background:**

Stroke-associated pneumonia (SAP) complicates 10–30% of acute ischemic stroke (AIS) cases and worsens outcomes. The C-reactive protein to albumin ratio (CAR) integrates inflammation and nutritional status and may serve as a prognostic biomarker. However, non-linear relationships and threshold effects for CAR in SAP prediction remain unclear.

**Methods:**

We retrospectively analyzed 1,595 consecutive AIS patients admitted between September 2016 and September 2022. CAR was calculated from admission CRP and albumin. Associations between CAR and SAP were assessed using multivariable logistic regression, generalized additive models (GAM), and two-piecewise regression to identify thresholds. Predictive performance was evaluated by ROC analysis and DeLong’s test.

**Results:**

A total of 1,595 patients were included (58.6% male; mean age 70.1 ± 12.2 years). The median admission NIHSS score was 3.0 (IQR 1.0–6.0). SAP occurred in 376 patients (23.6%). Log₂-CAR was strongly associated with SAP risk (P-trend < 0.0001), with the highest quartile showing a fully adjusted OR of 6.11 (95% CI: 3.63–10.27, *p* < 0.0001). This association was consistent across all subgroups (all P-interaction > 0.05). A non-linear threshold was identified at CAR ≈ 0.14; below this, the association was modest (OR = 1.22, 95% CI: 1.02–1.45), while above it, risk increased substantially (OR = 2.03, 95% CI: 1.70–2.42). The threshold model outperformed the linear model (*p* < 0.001). At the optimal cutoff of CAR = 0.18 (Youden’s index), CAR had superior predictive performance for SAP (AUC = 0.832, 95% CI: 0.807–0.858) compared to CRP (AUC = 0.827, *p* = 0.0002), A2DS2 (AUC = 0.764, *p* < 0.001), and WBC (AUC = 0.780, *p* = 0.0029). Adding CAR to baseline models improved prediction accuracy (all *p* < 0.05).

**Conclusion:**

CAR is a strong and independent predictor of stroke-associated pneumonia, outperforming traditional markers and improving risk prediction in acute stroke patients.

## 1 Introduction

Stroke remains a leading cause of death and disability globally (1, 2), with acute ischemic stroke (AIS) accounting for approximately 80% of all stroke cases (3). Despite revolutionary advances in acute stroke management—including tissue plasminogen activator therapy and mechanical thrombectomy (4–6)—post-stroke complications continue to substantially impact patient outcomes. Among these complications, stroke-associated pneumonia (SAP) represents one of the most frequent and clinically consequential, occurring in 10 to 30% of AIS patients depending on stroke severity and assessment methodology (3).

SAP imposes a considerable clinical and economic burden (7, 8). Patients who develop SAP experience prolonged hospital stays, elevated healthcare costs, higher rates of disability at discharge, and significantly increased mortality (7, 9). The 30-day mortality rate in SAP patients ranges from 25 to 40%, compared to 5 to 15% in AIS patients without pneumonia (7, 10). This substantial morbidity and mortality underscore the critical need for early identification of high-risk patients to enable targeted preventive interventions.

The pathophysiology of SAP is multifactorial, involving mechanical factors (impaired swallowing, aspiration), neurological factors (depressed consciousness, bulbar dysfunction), and crucially, immunological dysregulation (11–13). The acute stroke period is characterized by stroke-induced immunodepression syndrome (SIDS), a complex biphasic immune response featuring initial pro-inflammatory activation followed by profound systemic immunosuppression (14). This immunocompromised state creates a “window of vulnerability” for opportunistic infections, particularly in the respiratory tract (14).

Given this pathophysiological backdrop, inflammatory biomarkers have been extensively investigated as predictors of SAP. C-reactive protein (CRP) is the most widely studied marker, but demonstrates modest predictive accuracy (AUC typically 0.60–0.75) when used in isolation (15). Clinical prediction scores such as the A2DS2 perform better (AUC ~ 0.70–0.80) (16) but require clinical assessments that may not be immediately available.

The C-reactive protein to albumin ratio (CAR) represents a conceptually appealing composite biomarker that integrates two complementary pathophysiological dimensions (17, 18). CRP reflects the acute inflammatory burden, while albumin provides assessment of nutritional status, hepatic synthetic function, and chronic inflammatory stress. By combining these dimensions, CAR may capture the “immunometabolic vulnerability” that predisposes to post-stroke infections more comprehensively than either marker alone (14, 19, 20).

Recent studies have demonstrated associations between CAR and SAP in AIS populations. Most notably, Huang et al. reported that hypersensitive CRP-albumin ratio was associated with SAP (AUC = 0.810), with the highest quartile showing substantially elevated risk (21). However, this and other existing studies share a critical methodological limitation: they have examined CAR exclusively as a predictor with assumed linear exposure-response relationships. No previous investigation has employed advanced non-parametric statistical methods—such as generalized additive models (GAM) or segmented regression—to rigorously test whether the CAR-SAP relationship exhibits non-linearity or threshold effects.

This knowledge gap has profound clinical implications. If a threshold effect exists, it would define a critical tipping point—a specific CAR value beyond which infection risk escalates disproportionately. Identification of such an inflection point would enable more precise risk stratification, transforming CAR from a continuous predictor into a categorical screening tool with a clear, actionable cutoff value. This is fundamentally different from simply reporting odds ratios across quartiles, as it provides clinicians with an objective, quantitative criterion for immediate decision-making at the point of admission.

To address this critical gap in the literature, we hypothesized that the relationship between CAR and SAP risk is non-linear, with a clinically meaningful threshold effect that has not been previously identified. We conducted a large, single-center retrospective cohort study with three primary objectives: (1) to comprehensively characterize the association between CAR and SAP using multivariable logistic regression with rigorous covariate adjustment; (2) to employ GAM and two-piecewise regression to identify and validate potential threshold effects; and (3) to compare CAR’s predictive performance against established biomarkers and clinical prediction scores.

## 2 Materials and methods

### 2.1 Study design and setting

This retrospective cohort study was conducted at the Quzhou Affiliated Hospital of Wenzhou Medical University, a 2,000-bed tertiary care center and designated comprehensive stroke center. The study was approved by the hospital’s Institutional Ethics Committee (Approval No. 2023-151) and was conducted in accordance with the Declaration of Helsinki. Given the retrospective nature and use of de-identified data, the Ethics Committee granted a waiver of informed consent.

### 2.2 Study population

We screened all consecutive patients admitted with ischemic stroke between September 1, 2016, and September 30, 2022. Inclusion criteria were: (1) confirmed diagnosis of AIS according to WHO criteria with symptom onset within 24 h of presentation; (2) brain MRI with diffusion-weighted imaging performed within 48 h, demonstrating acute infarction confirmed by board-certified neuroradiologists; (3) blood samples obtained within 48 h of admission for CRP and albumin measurement; (4) complete clinical and laboratory data; (5) age ≥18 years.

**Figure 1:**
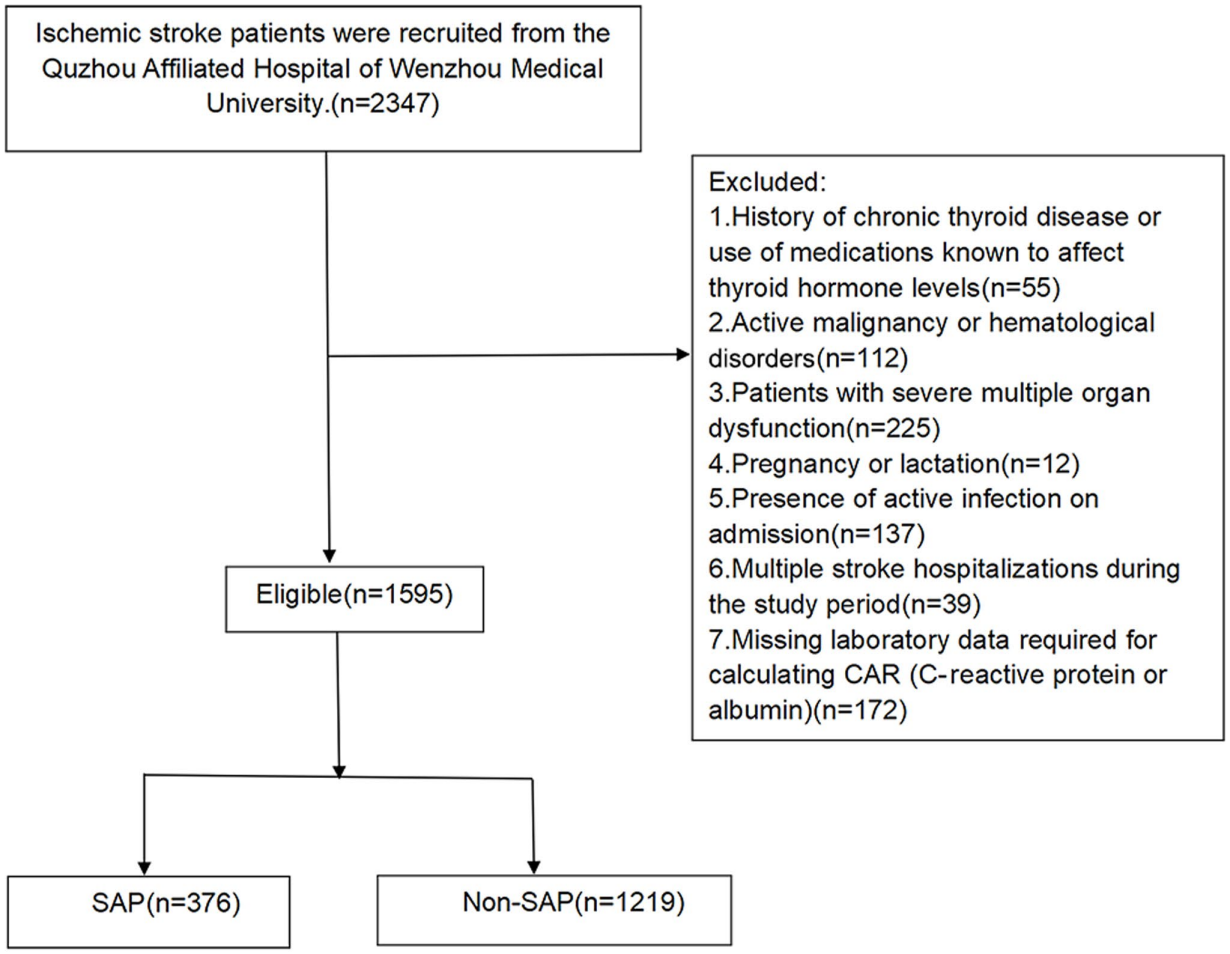
Patient selection flowchart demonstrating the derivation of the analytical cohort through systematic application of exclusion criteria. Flowchart showing patient selection from initial screening (*n* = 2,347) to final analytical cohort (*n* = 1,595) following systematic exclusion of 752 patients based on predefined criteria. Exclusion criteria included: chronic thyroid disease or thyroid-affecting medications (*n* = 55), active malignancy or hematological disorders (*n* = 112), severe multi-organ dysfunction (*n* = 225), pregnancy/lactation (*n* = 12), active infection at admission (*n* = 137), multiple stroke hospitalizations with retention of index admission only (*n* = 39), and missing laboratory data for CAR calculation (*n* = 172). The final cohort was stratified by clinical outcome into SAP (*n* = 376) and non-SAP (*n* = 1,219) groups. CAR, C-reactive protein to albumin ratio; SAP, stroke-associated pneumonia.

Exclusion criteria were systematically applied to minimize confounding: (1) chronic thyroid disease or medications affecting thyroid function (*n* = 55); (2) active malignancy or hematological disorders diagnosed within 5 years (*n* = 112); (3) severe multi-organ dysfunction at admission requiring intensive care (*n* = 225); (4) pregnancy or lactation (*n* = 12); (5) active infection at admission (*n* = 137), to ensure elevated CAR reflected stroke-related inflammation; (6) multiple stroke hospitalizations during the study period (*n* = 39), retaining only the index admission; (7) missing data for CAR calculation or essential covariates (*n* = 172). After exclusions, 1,595 patients constituted the analytical cohort (Figure 1).

### 2.3 Definition and diagnosis of SAP

SAP was diagnosed according to modified CDC criteria and Pneumonia in Stroke Consensus Group recommendations (22). Specifically, SAP required: (1) new or progressive pulmonary infiltrates on chest radiography or CT; plus (2) at least two of: fever (>38.0 °C) or hypothermia (<36.0 °C), leukocytosis (WBC > 12 × 10^9^/L) or leukopenia (<4 × 10^9^/L), purulent respiratory secretions, or new-onset cough/dyspnea. All SAP diagnoses were independently adjudicated by two experienced neurologists blinded to CAR values.

### 2.4 Data collection and laboratory measurements

Comprehensive baseline data were systematically extracted from electronic medical records. Demographic information included age and sex. Vascular risk factors included hypertension, diabetes mellitus, current smoking, atrial fibrillation, and COPD. Clinical assessments performed at admission included: (1) NIHSS for stroke severity (range 0–42) (23); (2) Dysphagia was evaluated using the Kubota Water Drinking Test (range 1–5) and defined as a score ≥ 3 points (24, 25); (3) Glasgow Coma Scale for consciousness level (range 3–15) (26). The need for nasal feeding and presence of consciousness disturbance were documented.

Blood samples were collected via venipuncture on the morning following admission (6:00–7:00 a.m., after overnight fasting) to standardize timing. Samples were immediately transported to the central laboratory, stored at 4 °C, and processed within 2 h. Laboratory tests included: CRP (high-sensitivity immunoturbidimetric assay), albumin (bromocresol green method), WBC count, neutrophil count, fasting glucose, HbA1c, lipid panel, uric acid, homocysteine, and creatinine. eGFR was calculated using the CKD-EPI equation (27). The A2DS2 clinical score was calculated according to published criteria (28).

CAR was calculated as CRP (mg/L) divided by albumin (g/L). To reduce skewness and facilitate statistical modeling, CAR values were log₂-transformed prior to analysis.

### 2.5 Statistical analysis

All statistical analyses were performed using R Studio (version 4.2.2) and EmpowerStats software (version 5.2). Continuous variables were assessed for normality using Shapiro–Wilk tests. Normally distributed variables are expressed as mean ± SD and compared using Student’s t-test. Non-normally distributed variables are expressed as median (IQR) and compared using Mann–Whitney U test. Categorical variables are presented as n (%) and compared using chi-square or Fisher’s exact test.

Log₂-transformed CAR was analyzed both as a continuous variable and categorized into quartiles (Q1-Q4). Multivariable logistic regression models with three adjustment levels were constructed: Model 1 (crude), Model 2 (age and sex adjusted), and Model 3 (fully adjusted for age, sex, smoking, hypertension, diabetes, atrial fibrillation, COPD, WBC, fasting glucose, eGFR, NIHSS, Dysphagia and GCS). Covariates were selected based on clinical relevance and univariate associations (*p* < 0.10). Variance inflation factors (VIF) were calculated to assess multicollinearity (VIF < 3 considered acceptable).

To identify non-linear relationships and threshold effects, we employed: (1) Generalized additive models (GAM) with penalized splines to flexibly model the CAR-SAP relationship without parametric assumptions, with smoothing parameter selection via generalized cross-validation; (2) Two-piecewise logistic regression using a recursive algorithm to identify the optimal inflection point (K) maximizing log-likelihood, estimating separate slopes below and above K, with 95% CIs obtained via 1,000 bootstrap iterations. Model superiority was tested using log-likelihood ratio tests.

Subgroup analyses examined the consistency of the CAR-SAP association across predefined strata: age (≤70 vs. > 70 years), gender, smoking status, hypertension, diabetes, atrial fibrillation, and COPD. Interaction testing was performed by including product terms in multivariable models.

ROC curve analysis evaluated predictive performance. AUC values with 95% CIs were compared using DeLong’s test for correlated curves. Optimal cutoffs were determined via Youden’s index. Sensitivity, specificity, PPV, NPV, and accuracy were calculated. Net reclassification improvement (NRI) and integrated discrimination improvement (IDI) quantified the incremental value of adding CAR to baseline models. All analyses used two-tailed tests with *p* < 0.05 considered significant.

## 3 Results

### 3.1 Patient selection and baseline characteristics

From 2,347 initially screened patients, 752 were excluded based on predefined criteria, resulting in a final analytical cohort of 1,595 patients (Figure 1). Among these, 376 (23.6%) developed SAP during hospitalization. The cohort had mean age 70.07 ± 12.19 years, with 41.38% female. Median admission NIHSS was 3.00 (IQR: 1.00–6.00).

Compared to non-SAP patients, those developing SAP were significantly older (74.49 ± 11.74 vs. 68.71 ± 12.00 years, *p* < 0.001), had higher prevalence of atrial fibrillation (35.11% vs. 11.50%, *p* < 0.001), nasal feeding requirement (38.03% vs. 1.56%, *p* < 0.001), consciousness disturbance (25.53% vs. 1.97%, *p* < 0.001), and COPD (15.69% vs. 4.02%, *p* < 0.001). SAP patients exhibited pronounced inflammatory activation: CAR (median 0.38 vs. 0.05, *p* < 0.001, 7.6-fold difference), CRP (13.62 vs. 2.00 mg/L, *p* < 0.001), WBC (9.24 vs. 6.55 × 10^9^/L, *p* < 0.001). Concurrently, they had lower albumin (36.21 vs. 38.44 g/L, *p* < 0.001), reduced eGFR (94.83 vs. 103.27 mL/min/1.73 m^2^, *p* < 0.001), and greater stroke severity (NIHSS: 5.00 vs. 2.00, *p* < 0.001) (Table 1).

**Table 1:** Baseline characteristics of the study population stratified by SAP and non-SAP groups.

Variables	Overall	Non-SAP	SAP	*p-*value
*N*	1,595	1,219	376	
Age (years)	70.07 ± 12.19	68.71 ± 12.00	74.49 ± 11.74	**<0.001**
Temperature (°C)	36.50 (36.20–36.80)	36.50 (36.20–36.70)	36.50 (36.20–36.80)	0.079
Gender, *n* (%)				0.260
Female	660 (41.38%)	495 (40.61%)	165 (43.88%)	
Male	935 (58.62%)	724 (59.39%)	211 (56.12%)	
Smoking, *n* (%)				0.593
No	1,028 (64.45%)	790 (64.81%)	238 (63.30%)	
Yes	567 (35.55%)	429 (35.19%)	138 (36.70%)	
Hypertension, *n* (%)				0.830
No	371 (23.26%)	282 (23.13%)	89 (23.67%)	
Yes	1,224 (76.74%)	937 (76.87%)	287 (76.33%)	
Diabetes mellitus, *n* (%)				0.947
No	1,037 (65.02%)	792 (64.97%)	245 (65.16%)	
Yes	558 (34.98%)	427 (35.03%)	131 (34.84%)	
Atrial fibrillation, *n* (%)				**<0.001**
No	1,321 (82.82%)	1,077 (88.35%)	244 (64.89%)	
Yes	274 (17.18%)	142 (11.50%)	132 (35.11%)	
Nasal feeding, *n* (%)				**<0.001**
No	1,433 (89.84%)	1,200 (98.44%)	233 (61.97%)	
Yes	162 (10.16%)	19 (1.56%)	143 (38.03%)	
Disturbance of consciousness, *n* (%)				**<0.001**
No	1,475 (92.48%)	1,195 (98.03%)	280 (74.47%)	
Yes	120 (7.52%)	24 (1.97%)	96 (25.53%)	
COPD, *n* (%)				**<0.001**
No	1,487 (93.23%)	1,170 (95.98%)	317 (84.31%)	
Yes	108 (6.77%)	49 (4.02%)	59 (15.69%)	
Laboratory parameters				
FPG (mg/dL)	101.81 (89.56–128.44)	99.83 (88.84–124.52)	108.48 (93.12–138.89)	**<0.001**
HbA1C (%)	6.00 (5.60–7.20)	6.00 (5.50–7.20)	6.10 (5.60–7.30)	0.338
TG (mg/dL)	111.60 (79.71–155.88)	117.80 (83.70–164.74)	93.00 (69.08–127.76)	**<0.001**
TC (mmol/L)	4.42 ± 1.10	4.47 ± 1.08	4.28 ± 1.14	**0.005**
LDLC (mmol/L)	2.89 ± 1.01	2.92 ± 1.00	2.80 ± 1.04	**0.024**
UA (umol/L)	313.70 (252.85–382.70)	315.40 (258.40–386.05)	303.75 (235.45–369.80)	**0.014**
HCY(umol/L)	15.30 (12.10–19.60)	15.00 (12.00–19.22)	16.30 (12.93–20.02)	**0.002**
CRP (mg/L)	2.90 (1.20–7.00)	2.00 (1.00–4.20)	13.62 (4.00–37.47)	**<0.001**
WBC (*109 /L)	7.00 (5.60–8.73)	6.55 (5.40–8.00)	9.24 (7.44–11.39)	**<0.001**
NTC (*109 /L)	4.55 (3.50–6.22)	4.18 (3.30–5.30)	7.00 (5.15–9.11)	**<0.001**
Albumin (g/L)	37.91 ± 4.05	38.44 ± 3.82	36.21 ± 4.33	**<0.001**
CAR	0.07 (0.03–0.19)	0.05 (0.03–0.11)	0.38 (0.11–1.05)	**<0.001**
EGFR (ml/min/1.73 m^2^)	101.28 ± 34.71	103.27 ± 33.41	94.83 ± 37.93	**<0.001**
Clinical characteristics				
NIHSS	3.00 (1.00–6.00)	2.00 (1.00–4.00)	5.00 (2.00–13.00)	**<0.001**
KWDT	1.00 (1.00–1.00)	1.00 (1.00–1.00)	1.00 (1.00–4.00)	**<0.001**
A2DS2	3.00 (2.00–4.00)	2.00 (2.00–3.00)	4.00 (3.00–7.00)	**<0.001**
GCS	15.00 (14.00–15.00)	15.00 (15.00–15.00)	14.00 (12.00–15.00)	<0.001

Data are presented as mean ± SD, median (interquartile range), or *n* (%). *p*-values were calculated using Student’s t-test, Mann–Whitney U test, or chi-square test as appropriate.

SAP, stroke-associated pneumonia; COPD, Chronic obstructive pulmonary disease; FPG, fasting plasma glucose; HbA1C, Glycated hemoglobin; TG, triglyceride; TC, total cholesterol; LDLC, low-density lipoprotein cholesterol; UA, uric acid; HCY, Homocysteine; CRP, C-reactive protein; WBC, white blood cell; NTC, neutrophil count; CAR, C-reactive protein to albumin ratio; EGFR, estimated glomerular filtration rate; NIHSS, National Institutes of Health Stroke Scale; KWDT, Kubota Water Swallowing Test; A2DS2, Age, Atrial fibrillation, Dysphagia, Sex, and Stroke Severity; GCS, Glasgow Coma Scale.

Bold values indicate statistical significance (*p* < 0.05).

### 3.2 CAR quartiles and SAP risk

SAP incidence increased dramatically across CAR quartiles: Q1 (6.55%), Q2 (9.09%), Q3 (15.15%), Q4 (63.73%), *p* < 0.001 (Table 2). Higher quartiles showed progressively greater prevalence of atrial fibrillation (7.81 to 27.96%), nasal feeding (0.50 to 29.97%), consciousness disturbance (0.76 to 22.42%), and COPD (4.28 to 14.11%), all *p* < 0.001. Laboratory trends included rising CRP (0.71 to 18.29 mg/L), WBC (6.25 to 8.80 × 10^9^/L), neutrophils (3.85 to 6.55 × 10^9^/L), and declining albumin (39.31 to 36.10 g/L), all *p* < 0.001.

**Table 2:** Baseline characteristics of patients stratified by log₂-CAR quartiles.

CAR Log2 quartile	Q1	Q2	Q3	Q4	*p*-value
*N*	397	396	396	397	
SAP	26 (6.55%)	36 (9.09%)	60 (15.15%)	253 (63.73%)	**<0.001**
Age (years)	67.36 ± 11.56	68.85 ± 11.96	70.58 ± 12.31	73.42 ± 12.16	**<0.001**
Temperature (°C)	36.50 (36.10–36.70)	36.50 (36.20–36.70)	36.50 (36.20–36.80)	36.50 (36.20–36.80)	**<0.001**
Gender, *n* (%)					0.459
Female	159 (40.05%)	157 (39.65%)	177 (44.70%)	163 (41.06%)	
Male	238 (59.95%)	239 (60.35%)	219 (55.30%)	234 (58.94%)	
Smoking, *n* (%)					**0.023**
No	259 (65.24%)	236 (59.60%)	277 (69.95%)	252 (63.48%)	
Yes	138 (34.76%)	160 (40.40%)	119 (30.05%)	145 (36.52%)	
Hypertension, *n* (%)					0.104
No	96 (24.18%)	74 (18.69%)	100 (25.25%)	98 (24.69%)	
Yes	301 (75.82%)	322 (81.31%)	296 (74.75%)	299 (75.31%)	
Diabetes, *n* (%)					0.434
No	259 (65.24%)	270 (68.18%)	249 (62.88%)	254 (63.98%)	
Yes	138 (34.76%)	126 (31.82%)	147 (37.12%)	143 (36.02%)	
Atrial fibrillation, *n* (%)					**<0.001**
No	366 (92.19%)	348 (87.88%)	314 (79.29%)	286 (72.04%)	
Yes	31 (7.81%)	48 (12.12%)	82 (20.71%)	111 (27.96%)	
Nasal feeding, *n* (%)					**<0.001**
No	395 (99.50%)	385 (97.22%)	367 (92.68%)	278 (70.03%)	
Yes	2 (0.50%)	11 (2.78%)	29 (7.32%)	119 (29.97%)	
Disturbance of consciousness, *n* (%)					**<0.001**
No	394 (99.24%)	388 (97.98%)	376 (94.95%)	308 (77.58%)	
Yes	3 (0.76%)	8 (2.02%)	20 (5.05%)	89 (22.42%)	
COPD, *n* (%)					**<0.001**
No	380 (95.72%)	377 (95.20%)	381 (96.21%)	341 (85.89%)	
Yes	17 (4.28%)	19 (4.80%)	15 (3.79%)	56 (14.11%)	
Laboratory parameters					
FPG (mg/dL)	99.29 (88.48–120.55)	99.65 (89.47–122.54)	101.18 (89.74–131.50)	108.12 (90.64–139.29)	**<0.001**
HbA1C (%)	6.00 (5.50–7.30)	6.00 (5.60–7.10)	6.10 (5.60–7.20)	6.00 (5.60–7.30)	0.957
TG (mg/dL)	116.91 (82.37–162.08)	118.68 (83.92–168.28)	113.81 (82.37–160.53)	94.77 (68.20–131.97)	**<0.001**
TC (mmol/L)	4.33 ± 1.06	4.53 ± 1.00	4.54 ± 1.17	4.29 ± 1.14	**<0.001**
LDLC (mmol/L)	2.78 ± 0.96	2.98 ± 0.93	3.00 ± 1.05	2.79 ± 1.07	**<0.001**
UA (umol/L)	308.00 (255.00–386.00)	315.00 (257.73–386.55)	319.65 (258.03–385.40)	312.00 (244.60–375.10)	0.675
HCY (umol/L)	14.90 (11.50–19.00)	14.60 (11.89–18.50)	15.70 (12.33–20.00)	16.30 (12.69–20.30)	**<0.001**
CRP (mg/L)	0.71 (0.45–1.00)	2.00 (1.60–2.25)	4.01 (3.40–5.23)	18.29 (11.09–40.46)	**<0.001**
WBC (*109/L)	6.25 (5.10–7.51)	6.69 (5.50–8.00)	7.10 (5.70–8.76)	8.80 (6.97–11.21)	**<0.001**
NTC (*109 /L)	3.85 (3.12–4.87)	4.20 (3.30–5.21)	4.64 (3.60–6.08)	6.55 (4.68–9.05)	**<0.001**
Albumin (g/L)	39.31 ± 4.01	38.56 ± 3.42	37.68 ± 3.72	36.10 ± 4.31	**<0.001**
EGFR (ml/min/1.73 m^2^)	106.32 ± 32.49	103.05 ± 32.07	100.82 ± 35.74	95.20 ± 37.42	**<0.001**
Clinical characteristics					
NIHSS	2.00 (1.00–4.00)	2.00 (1.00–5.00)	2.00 (1.00–5.00)	4.00 (2.00–12.00)	**<0.001**
KWDT	1.00 (1.00–1.00)	1.00 (1.00–1.00)	1.00 (1.00–1.00)	1.00 (1.00–4.00)	**<0.001**
A2DS2	2.00 (2.00–3.00)	2.00 (2.00–4.00)	3.00 (2.00–4.00)	4.00 (2.00–6.00)	**<0.001**
GCS	15.00 (15.00–15.00)	15.00 (15.00–15.00)	15.00 (14.00–15.00)	14.00 (12.00–15.00)	**<0.001**

Data are presented as mean ± SD, median (interquartile range), or *n* (%). *p*-values were calculated using one-way ANOVA, Kruskal-Wallis H test, or chi-square test as appropriate.

CAR quartiles: Q1 (lowest), Q2, Q3, Q4 (highest). The log₂-transformed CAR values were divided into quartiles for stratification analysis. Corresponding CAR value ranges: Q1 (CAR < 0.031), Q2 (0.031 ≤ CAR < 0.073), Q3 (0.073 ≤ CAR < 0.180), Q4 (CAR ≥ 0.180). Notably, the Youden index–derived optimal diagnostic threshold of 0.180 coincides with the lower boundary of Q4, consistent with the markedly elevated SAP risk observed in this quartile.

CAR, C-reactive protein to albumin ratio; SAP, stroke-associated pneumonia; COPD, Chronic obstructive pulmonary disease; FPG, fasting plasma glucose; HbA1C, Glycated hemoglobin; TG, triglyceride; TC, total cholesterol; LDLC, low-density lipoprotein cholesterol; HCY, Homocysteine; UA, uric acid; CRP, C-reactive protein; WBC, white blood cell; NTC, Neutrophil Count; EGFR, estimated glomerular filtration rate; NIHSS, National Institutes of Health Stroke Scale; KWDT, Kubota Water Swallowing Test; A2DS2, Age, Atrial fibrillation, Dysphagia, Sex, and Stroke Severity; GCS, Glasgow Coma Scale.

Bold values indicate statistical significance (*p* < 0.05).

### 3.3 Multivariable logistic regression analysis

Unadjusted ORs for SAP by quartile were: Q2: 1.43 (0.84–2.41), Q3: 2.55 (1.57–4.13, *p* = 0.0001), Q4: 25.07 (16.03–39.21, *p* < 0.0001). After age- and sex-adjustment (Model 2), the Q4 OR remained markedly elevated: Q2: 1.36 (0.80–2.31), Q3: 2.31 (1.42–3.76, *p* = 0.0007), Q4: 22.29 (14.20–34.99, *p* ≤ 0.0001). After full adjustment for all predefined covariates (Model 3), associations remained highly significant: Q2: 1.11 (0.63–1.95, *p* = 0.730), Q3: 1.19 (0.69–2.06, *p* = 0.539), Q4: 6.11 (3.63–10.27, *p* ≤ 0.0001). P-trend was < 0.0001 across all three models, confirming a robust dose–response relationship between CAR and SAP risk (Table 3).

**Table 3:** Multivariable logistic regression analysis of the association between log₂-transformed CAR and stroke-associated pneumonia.

Exposure	Crude model (Model 1)			Partially adjusted model (Model 2)			Fully adjusted model (Model 3)		
	OR (95% CI)	*p*-value	P-trend	OR (95% CI)	*p*-value	P-trend	OR (95% CI)	*p*-value	P-trend
Log₂-CAR	2.12 (1.95, 2.31)	<0.0001	<0.0001	2.08 (1.91, 2.26)	<0.0001	<0.0001	1.61 (1.46, 1.77)	<0.0001	<0.0001
Log₂-CAR quartile									
Q1	1.0	1.0		1.0	1.0		1.0	1.0	
Q2	1.43 (0.84, 2.41)	1.43 (0.84, 2.41)	0.1843	1.36 (0.80, 2.31)	1.36 (0.80, 2.31)	0.2486	1.11 (0.632, 1.95)	1.11 (0.632, 1.95)	0.7296
Q3	2.55 (1.57, 4.13)	2.55 (1.57, 4.13)	**0.0001**	2.31 (1.42, 3.76)	2.31 (1.42, 3.76)	**0.0007**	1.19 (0.69, 2.06)	1.19 (0.69, 2.06)	0.5393
Q4	25.07 (16.03, 39.21)	25.07 (16.03, 39.21)	**<0.0001**	22.29 (14.20, 34.99)	22.29 (14.20, 34.99)	**<0.0001**	6.11 (3.63, 10.27)	6.11 (3.63, 10.27)	**<0.0001**
Log_2_-CAR quartile continuous	3.57 (3.06, 4.16)	3.57 (3.06, 4.16)	**<0.0001**	3.42 (2.93, 3.99)	3.42 (2.93, 3.99)	**<0.0001**	1.96 (1.65, 2.34)	1.96 (1.65, 2.34)	**<0.0001**

Model 1: Unadjusted.

Model 2: Adjusted for age and sex.

Model 3: Fully adjusted for age, sex, smoking status, hypertension, diabetes mellitus, atrial fibrillation, chronic obstructive pulmonary disease, dysphagia, National Institutes of Health Stroke Scale score, Glasgow Coma Scale score, white blood cell count, fasting plasma glucose, and estimated glomerular filtration rate.

The log₂-transformed CAR values were analyzed both as a continuous variable and categorized into quartiles (Q1–Q4). Q1 represents the lowest quartile and serves as the reference group. P-trend values were calculated by treating quartiles as ordinal variables. Quartile boundaries (CAR values): Q1 (CAR < 0.031), Q2 (0.031 ≤ CAR < 0.073), Q3 (0.073 ≤ CAR < 0.180), Q4 (CAR ≥ 0.180).

CAR, C-reactive protein to albumin ratio; SAP, stroke-associated pneumonia; OR, odds ratio; CI, confidence interval.

Bold values indicate statistical significance (*p* < 0.05).

As a continuous variable, each doubling of CAR (1-unit increase in log₂-CAR) conferred increased SAP risk: crude OR = 3.57 (3.06–4.16), fully adjusted OR = 1.96 (1.65–2.34), all *p* < 0.0001. VIF analysis confirmed acceptable multicollinearity across all 14 covariates in Model 3 (all VIF ≤ 3.0, mean VIF = 1.55, range: 1.1–2.6). The highest VIF values were observed for NIHSS (2.6) and GCS (2.5), reflecting their conceptual overlap as measures of neurological impairment, while the primary exposure log₂-CAR showed low multicollinearity (VIF = 1.4). Full covariate-specific VIF values are presented in Table 4.

**Table 4:** Variance inflation factors (VIFs) for covariates included in the multivariable logistic regression model.

Variable	Description	VIF	Interpretation
log2_CAR	Log₂-transformed CAR (primary exposure)	1.4	Low
NIHSS score	NIH Stroke Scale score	2.6	Low
AF	Atrial fibrillation	1.2	Low
Hypertension	Hypertension	1.1	Low
FPG	Fast Plasma Glucose(mg/dL)	1.5	Low
Age	Age (years)	1.3	Low
WBC	White blood cell count (*10^9^/L)	1.3	Low
Gender	Gender (male = 1)	1.7	Low
Smoking	Current smoking status	1.7	Low
EGFR	Estimated glomerular filtration rate (ml/min/1.73 m^2^)	1.2	Low
COPD	Chronic obstructive pulmonary disease	1.1	Low
Diabetes	Diabetes mellitus	1.4	Low
GCS score	Glasgow Coma Scale	2.5	Low
Dysphagia	Kubota Water Swallowing Test≥3	1.7	Low

VIF values assess multicollinearity among independent variables in Model 3 (fully adjusted model from Table 3).

Interpretation criteria: VIF < 5 indicates low multicollinearity (acceptable); VIF 5–10 indicates moderate multicollinearity (caution); VIF ≥ 10 indicates high multicollinearity (problematic).

All VIF values in the current model are below 3.0, indicating no significant multicollinearity. Mean VIF = 1.55 (range: 1.1–2.6), confirming excellent model quality and reliable parameter estimates.

VIF, variance inflation factor; CAR, C-reactive protein to albumin ratio; NIHSS, National Institutes of Health Stroke Scale; AF, atrial fibrillation; FPG, fasting plasma glucose; WBC, white blood cell count; EGFR, estimated glomerular filtration rate; COPD, chronic obstructive pulmonary disease; COPD, chronic obstructive pulmonary disease; GCS, Glasgow Coma Scale.

### 3.4 Subgroup analysis and interaction testing

**Figure 2:**
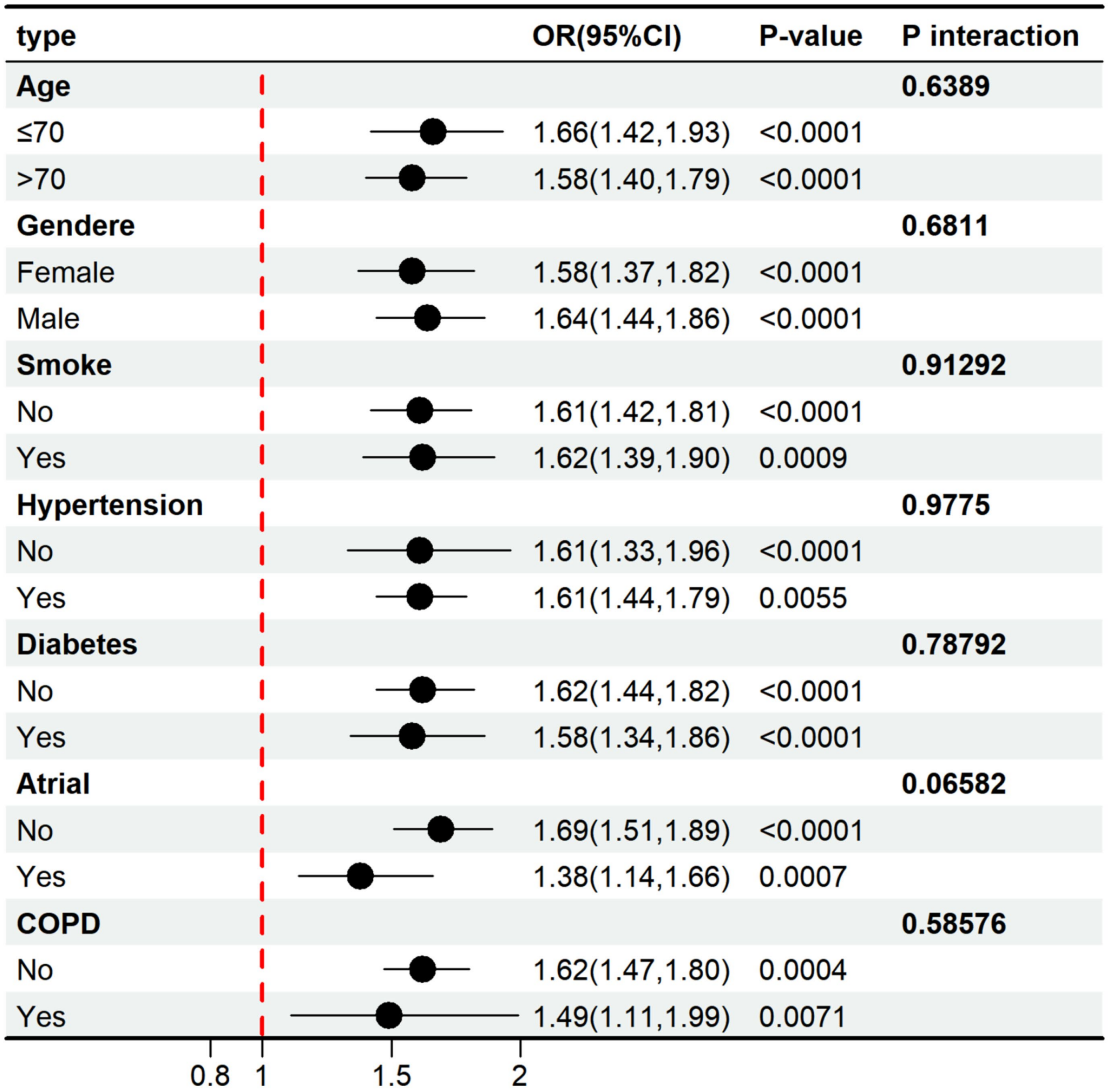
Subgroup analysis demonstrating the robust consistency of the CAR-SAP association across diverse patient populations. Forest plot showing ORs and 95% CI for the association between log₂-transformed CAR and SAP, stratified by seven clinical variables: age (≤70 vs. >70 years), gender (female vs. male), smoking status (non-smoker vs. smoker), hypertension (no vs. yes), diabetes mellitus (no vs. yes), atrial fibrillation (no vs. yes), and COPD (no vs. yes). Each point estimate is represented by a black circle, with horizontal lines indicating 95% CI. The vertical dashed line at OR = 1.0 represents the null effect. *p* values for interaction are shown for each stratifying variable. OR, odds ratio; CI, confidence interval; COPD, chronic obstructive pulmonary disease.

The CAR-SAP association remained consistent across all examined subgroups (Figure 2). Adjusted ORs ranged from 1.38 (atrial fibrillation present) to 1.69 (atrial fibrillation absent), with all subgroups showing statistically significant positive associations (all *p* < 0.01). Formal interaction testing revealed no significant effect modification: age (*p* = 0.6389), gender (*p* = 0.6811), smoking (*p* = 0.9129), hypertension (*p* = 0.9775), diabetes (*p* = 0.7879), atrial fibrillation (*p* = 0.0658), and COPD (*p* = 0.5858). The consistent effect across subgroups confirms the robustness and generalizability of the CAR-SAP relationship.

### 3.5 Predictive performance and model comparisons

CAR demonstrated excellent predictive performance: AUC = 0.832 (95% CI: 0.807–0.857), compared to CRP (AUC = 0.827; DeLong’s *p* = 0.0002), WBC (AUC = 0.780; *p* = 0.0029), NTC (AUC = 0.803; *p* = 0.087), and A2DS2 (AUC = 0.764; *p* ≤ 0.001). CAR was statistically superior to CRP, WBC, and A2DS2, but not significantly different from NTC. At the optimal cutoff of CAR = 0.180 (determined by Youden’s index), sensitivity was 68.6%, specificity 87.5%, PPV 62.9%, NPV 90.0%, and accuracy 83.1% (Table 5). Detailed pairwise AUC comparisons are presented in Table 6.

**Table 5:** Comparison of diagnostic performance of CAR, CRP, WBC NTC and A2DS2 for predicting stroke-associated pneumonia (SAP).

Biomarker	AUC	95% CI	Cutoff	Sensitivity (%)	Specificity (%)	PPV (%)	NPV (%)	Accuracy (%)
CAR	0.832	0.807–0.857	0.180	68.6	87.5	62.9	90.0	83.1
CRP	0.827	0.801–0.853	7.0	66.8	87.1	61.5	89.5	82.3
WBC	0.780	0.752–0.809	8.23	66.8	78.9	49.4	88.5	76.1
NTC	0.803	0.776–0.831	6.20	63.6	86.4	59.0	88.5	81.0
A2DS2	0.764	0.735–0.793	4.5	48.1	91.5	63.5	85.1	81.3

ROC curve analysis was performed to evaluate the diagnostic performance of inflammatory biomarkers and the clinical A2DS2 score for predicting stroke-associated pneumonia (SAP). The optimal cutoff value for each predictor was determined using the Youden index (maximizing sensitivity + specificity − 1). The area under the curve (AUC) was calculated with 95% confidence intervals (CIs) estimated using bootstrap resampling (1,000 iterations).

AUC, area under the receiver operating characteristic curve; CI, confidence interval; PPV, positive predictive value; NPV, negative predictive value; CAR, C-reactive protein to albumin ratio; CRP, C-reactive protein; WBC, white blood cell count; NTC, neutrophil count; A2DS2, Age, Atrial fibrillation, Dysphagia, Sex, and Stroke Severity; SAP, stroke-associated pneumonia. Optimal cutoff values were determined using Youden’s index.

**Table 6:** Results of statistical tests for pairwise comparisons.

Comparison (A vs B)	AUC (A)	AUC (B)	*p* value* (DeLong test)	Conclusion
CAR vs. A2DS2	0.832	0.764	**<0.001**	Superior
CAR vs. CRP	0.832	0.827	**0.0002**	Superior
CAR vs. NTC	0.832	0.803	0.087	Not Significant
CAR vs. WBC	0.832	0.780	**0.0029**	Superior

*p values were derived from DeLong’s test for comparing two ROC curves.

*p < 0.05 for comparison between the baseline model and the model incorporating CAR.

AUC, area under the receiver operating characteristic curve; CI, confidence interval; CAR, C-reactive protein to albumin ratio; CRP, C-reactive protein; WBC, white blood cell coun**t**; NTC, neutrophil count; A2DS2, Age, Atrial fibrillation, Dysphagia, Sex, and Stroke Severity; SAP, stroke-associated pneumonia.

Bold values indicate statistical significance (*p* < 0.05).

**Figure 3:**
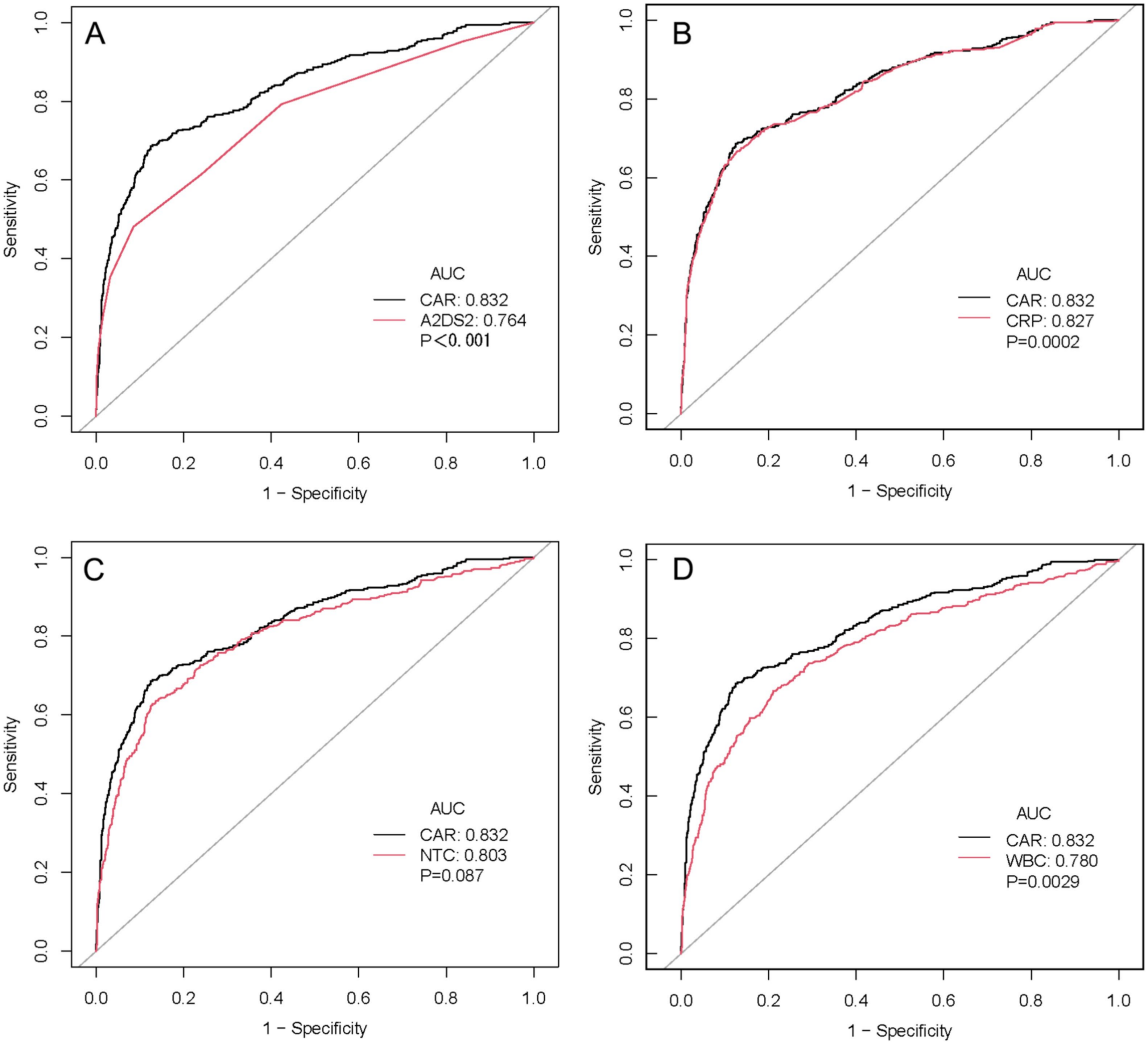
ROC curve comparison of CAR versus alternative predictors for SAP. Pairwise ROC curve comparisons between CAR (black line) and **(A)** A2DS2 score (AUC: 0.832 vs. 0.764, *p* < 0.001), **(B)** CRP (AUC: 0.832 vs. 0.827, *p* = 0.0002), **(C)** neutrophil count (AUC: 0.832 vs. 0.803, *p* = 0.087), and **(D)** white blood cell count (AUC: 0.832 vs. 0.780, *p* = 0.0029). The grey diagonal line represents no discrimination (AUC = 0.5). *p*-values were derived from DeLong’s test for correlated ROC curves. ROC, receiver operating characteristic; AUC, area under the curve; CAR, C-reactive protein to albumin ratio; A2DS2, age, atrial fibrillation, dysphagia, sex, stroke severity score; CRP, C-reactive protein; WBC, white blood cell; NTC, neutrophil count.

DeLong’s test revealed CAR significantly superior to A2DS2 (*p* < 0.001), CRP (*p* = 0.0002), and WBC (*p* = 0.0029), but not different from NTC (*p* = 0.087) (Table 6, Figure 3). The high NPV (90.0%) indicates CAR’s particular utility in ruling out SAP in low-risk patients.

### 3.6 Incremental predictive value of CAR

**Figure 4:**
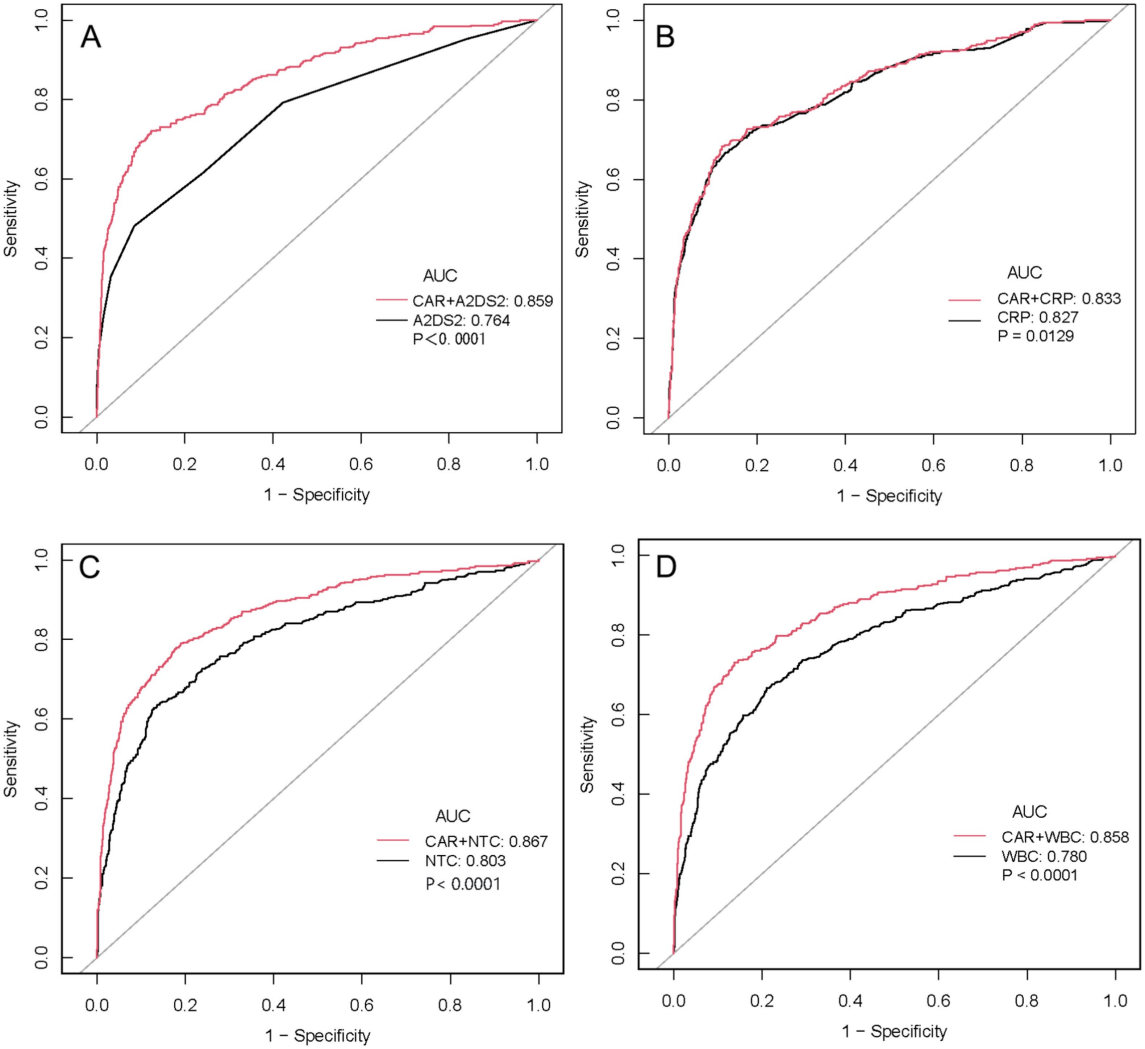
Incremental predictive value of CAR when added to established baseline models for SAP prediction. Comparison of ROC curves for baseline predictors alone (black line) versus baseline predictors combined with CAR (red line). **(A)** A2DS2 score vs. A2DS2 + CAR: combined model AUC = 0.859 vs. A2DS2 alone AUC = 0.764 (*p* < 0.0001). **(B)** CRP vs. CRP + CAR: combined model AUC = 0.833 vs. CRP alone AUC = 0.827 (*p* = 0.0129). **(C)** Neutrophil count vs. Neutrophil count + CAR: combined model AUC = 0.867 vs. Neutrophil count alone AUC = 0.803 (*p* < 0.0001). **(D)** White blood cell count vs. White blood cell count + CAR: combined model AUC = 0.858 vs. White blood cell count alone AUC = 0.780 (*p* < 0.0001). The grey diagonal line represents random chance (AUC = 0.5). All *p*-values were calculated using DeLong’s test. ROC, receiver operating characteristic; AUC, area under the curve; CAR, C-reactive protein to albumin ratio; A2DS2, age, atrial fibrillation, dysphagia, sex, stroke severity score; CRP, C-reactive protein; WBC, white blood cell; NTC, neutrophil count.

Adding CAR to established baseline models significantly improved prediction across all scenarios (Figure 4). When added to A2DS2, AUC increased from 0.764 to 0.859 (ΔAUC = +0.095, *p* < 0.0001), representing a 12.4% relative improvement. Significant improvements were also observed when CAR was added to CRP (0.827 → 0.833, *p* = 0.0129), NTC (0.803 → 0.867, *p* < 0.0001), and WBC (0.780 → 0.858, *p* < 0.0001). These findings demonstrate that CAR provides meaningful incremental predictive value across diverse baseline models (Table 7).

**Table 7:** Performance and incremental value of models with and without CAR for SAP prediction.

Predictor(s) in baseline model	AUC (Baseline)	AUC (Baseline + CAR)	NRI (95%CI)	IDI (95%CI)
A2DS2	0.764	0.859	0.2008 (0.1563 to 0.2453)*	0.2008 (0.1563 to 0.2453)*
CRP	0.827	0.833	0.0242 (0.0045 to 0.0438)*	0.0242 (0.0045 to 0.0438)*
WBC	0.780	0.858	0.1377 (0.0929 to 0.1824)*	0.1377 (0.0928 to 0.1825)*
NTC	0.803	0.7867	0.1009 (0.0622 to 0.1397)*	0.1009 (0.0621 to 0.1397)*

The area under the ROC curve (AUC) for models with and without the CAR was compared using DeLong’s test (*p* < 0.05). The Net Reclassification Improvement (NRI) and Integrated Discrimination Improvement (IDI) with 95% CIs were calculated to quantify the improvement in predictive performance. An asterisk (*) denotes a statistically significant change (95% CI does not include zero).

AUC, area under the receiver operating characteristic curve; CI, confidence interval; NRI, Net Reclassification Improvement; IDI, Integrated Discrimination Improvement; CAR, C-reactive protein to albumin ratio; CRP, C-reactive protein; WBC, white blood cell count; NTC, neutrophil count; A2DS2, Age, Atrial fibrillation, Dysphagia, Sex, and Stroke Severity; SAP, stroke-associated pneumonia.

### 3.7 Non-linear relationship and threshold effect

**Figure 5:**
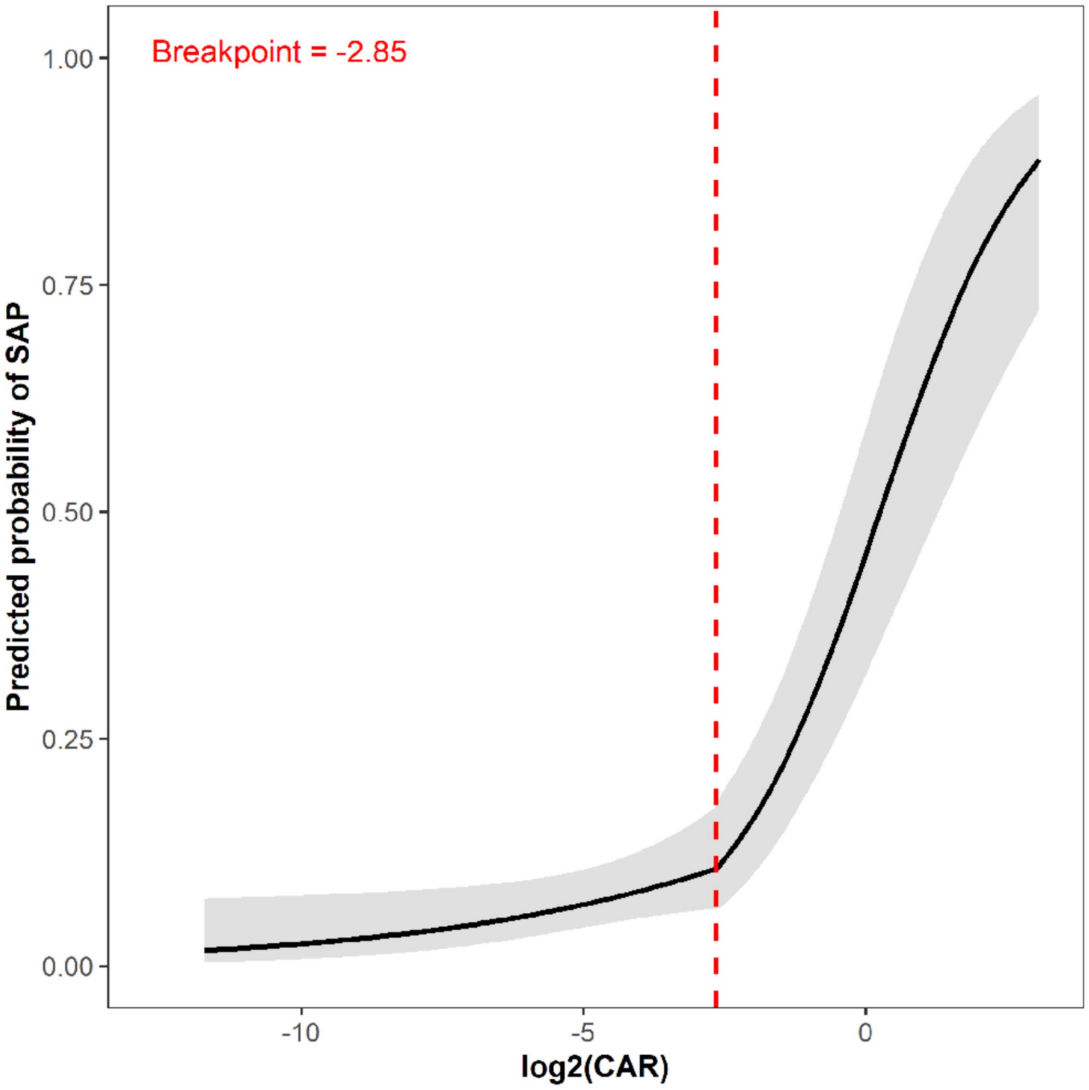
Non-linear relationship between log_2_-transformed CAR and predicted probability of SAP, revealing a threshold effect at the inflection point. The GAM smooth curve (black solid line) visualizes the relationship between log_2_-CAR and the predicted probability of SAP, with the grey shaded area representing 95% CI. The red dashed vertical line marks the inflection point at log_2_-CAR = −2.85 (corresponding to CAR ≈ 0.18), as identified by two-piecewise logistic regression analysis (see Table 8 for detailed model comparisons and odds ratios). The relationship shows a change in slope around this inflection point. GAM, generalized additive model.

GAM analysis revealed a clear non-linear, threshold-dependent relationship between log₂-CAR and SAP risk (Figure 5). The curve was relatively flat at low CAR values with predicted SAP probability remaining below 5%. At log₂-CAR ≈ − 2.85 (CAR ≈ 0.14), the curve began to steepen markedly, with risk escalating dramatically above this inflection point.

Two-piecewise logistic regression formally confirmed the threshold effect (Table 8). Linear model (Model I) showed: OR = 1.60 (1.45–1.76, *p* < 0.0001). Threshold model (Model II) identified inflection point at log₂-CAR = −2.85. Below threshold: OR = 1.22 (1.02–1.45, *p* = 0.031). Above threshold: OR = 2.03 (1.70–2.42, *p* < 0.0001). The difference between segments was statistically significant (OR = 1.67, 95% CI: 1.23–2.25, *p* < 0.001), and log-likelihood ratio test strongly confirmed threshold model superiority (*p* < 0.001).

**Table 8:** Multivariable logistic regression models evaluating the linear and two-piecewise associations between log₂-transformed C-reactive protein to albumin ratio (CAR) and the risk of stroke-associated pneumonia (SAP).

SAP outcome	Adjusted OR (95% CI)	*p-*value
log₂-CAR (Model I)		
Linear association (log₂-CAR)	1.60 (1.45–1.76)	**< 0.0001**
log₂-CAR (Model II)		
Inflection point	−2.85	
log₂-CAR < −2.85	1.22 (1.02, 1.45)	**0.0307**
log₂-CAR ≥ − 2.85	2.03 (1.70, 2.42)	**< 0.0001**
Difference (Segment 2–1)	1.67 (1.23, 2.25)	**0.000**
Log likelihood ratio		**< 0.001**

Model I assumes a linear effect, while Model II incorporates a threshold at the inflection point (K = −2.85). Estimates are adjusted for age, sex, smoking status, hypertension, diabetes mellitus, atrial fibrillation, chronic obstructive pulmonary disease, white blood cell count, fasting plasma glucose, estimated glomerular filtration rate, baseline NIHSS score, Glasgow Coma Scale and dysphagia.

CAR, C-reactive protein to albumin ratio; SAP, stroke-associated pneumonia; OR, odds ratio; CI, confidence interval.

Bold values indicate statistical significance (*p* < 0.05).

It should be noted that two distinct but complementary CAR threshold values are reported in this study. The mathematically derived inflection point from the two-piecewise regression model is log₂-CAR = −2.85, corresponding to a CAR value of approximately 0.14. This value represents the point of maximum change in slope of the CAR-SAP association and is obtained by maximizing the log-likelihood function. In contrast, the clinically recommended cutoff of CAR = 0.180 was derived independently from ROC curve analysis using Youden’s index (maximizing sensitivity + specificity – 1). These two methods address complementary questions: the piecewise regression identifies the biological tipping point where the exposure-response gradient changes most steeply, while the Youden index identifies the threshold that optimizes diagnostic discrimination. We chose to present 0.18 as the primary clinical recommendation because it was derived from the ROC analysis optimized for clinical decision-making (sensitivity 68.6%, specificity 87.5%), and it aligns precisely with the lower boundary of the highest-risk Q4 quartile (CAR ≥ 0.180). The slight discrepancy between the two values (0.14 vs. 0.18) likely reflects the different statistical objectives of each method: the regression inflection point captures where risk begins to accelerate, whereas the Youden threshold captures where the overall balance of sensitivity and specificity is optimized for SAP detection.

## 4 Discussion

In this large retrospective cohort of 1,595 AIS patients, we demonstrate four principal findings: (1) CAR exhibits a strong, dose–response relationship with SAP risk, with the highest quartile showing six-fold elevated odds after comprehensive covariate adjustment; (2) This association remains robust and consistent across diverse demographic and clinical subgroups, with no significant effect modification detected; (3) CAR demonstrates a non-linear, threshold-dependent relationship with a critical inflection point at log₂-CAR = −2.85 (CAR ≈ 0.14), confirmed by multiple statistical approaches; (4) CAR provides superior predictive performance compared to individual inflammatory markers and clinical scores, with significant incremental value when added to existing models.

Importantly, this represents the first study to employ advanced non-parametric statistical methods (GAM and two-piecewise regression) to rigorously characterize the mathematical form of the CAR-SAP relationship, identifying a clinically actionable threshold that transforms CAR from a continuous predictor into a categorical risk stratification tool.

The threshold effect we identified at CAR ≈ 0.18 provides critical mechanistic insights into post-stroke immune dysregulation. CAR is not merely a mathematical ratio but a composite representation of two interrelated pathophysiological processes that converge to create “immunometabolic vulnerability.”

The numerator—elevated CRP—reflects systemic inflammatory activation. Following acute ischemic brain injury, damaged neurons and glial cells release damage-associated molecular patterns (DAMPs) (29), activating pattern recognition receptors and triggering a cytokine cascade dominated by IL-1β, IL-6, and TNF-*α* (30). This pro-inflammatory milieu stimulates hepatic CRP synthesis (31). However, the same cytokine storm simultaneously activates the hypothalamic–pituitary–adrenal axis and sympathetic nervous system as part of SIDS (14, 32). This neuroendocrine response initially serves as compensatory anti-inflammation but comes at the cost of systemic immunosuppression—characterized by lymphocyte apoptosis, impaired T-cell proliferation, reduced NK cell activity, and functional deactivation of peripheral monocytes (33). This creates a paradoxical state where high CRP coexists with impaired antimicrobial defenses.

The denominator—decreased albumin—represents a complex, multifaceted process. In acute stroke, hypoalbuminemia results from: (1) Suppressed hepatic synthesis, as IL-6 and IL-1 redirect hepatic protein synthesis toward acute-phase reactants (34); (2) Increased vascular permeability, allowing albumin extravasation into interstitial compartments (35); (3) Accelerated catabolism, with albumin serving as an amino acid reservoir; (4) Pre-existing nutritional depletion in elderly patients, further exacerbated by acute illness.

Critically, hypoalbuminemia is not a passive marker but an active contributor to immune dysfunction. Albumin binds and neutralizes endotoxins, scavenges reactive oxygen species, modulates neutrophil adhesion, and influences cytokine bioavailability (36–38). Low albumin levels therefore directly impair these protective functions, reducing opsonizing antibodies, complement activation, and antimicrobial peptide expression.

The threshold at CAR = 0.18 likely represents the point where these dual processes—pro-inflammatory immunosuppression (high CRP) and metabolic depletion with loss of immunoprotective capacity (low albumin)—reach critical confluence. Below this threshold, compensatory mechanisms remain relatively intact. Above it, they fail catastrophically, creating a “perfect storm” where the immune system can neither mount effective antimicrobial responses nor maintain the metabolic infrastructure required for immunocompetence. This explains the dramatic risk escalation observed above the threshold in our GAM and piecewise analyses.

Our findings build upon but fundamentally extend previous work on CAR and SAP. Huang et al. demonstrated that hs-CRP-albumin ratio was associated with SAP (AUC = 0.810), with highest quartile showing OR = 17.72 (21). However, their analysis assumed linear relationships and did not test for non-linearity. Our identification of a threshold at CAR = 0.18 provides more nuanced understanding: the extraordinarily high OR in their Q4 likely reflects patients both near and far above the threshold, with the latter driving extreme risk.

Our AUC (0.832) is comparable to Huang’s (0.810) despite using standard CRP rather than hs-CRP, suggesting the ratio’s predictive value derives primarily from integrating inflammation and nutrition rather than CRP measurement sensitivity. This has important accessibility implications, as standard CRP is universally available.

An elevated CRP/albumin ratio was significantly associated with increased in-hospital mortality in patients with intracerebral hemorrhage (ICH), with each unit increment corresponding to a 15.3% rise in mortality risk (39). These findings across stroke subtypes suggest CAR reflects a fundamental vulnerability phenotype. Our threshold identification provides a unifying framework: CAR ≈ 0.18 may represent a generalizable “immunometabolic inflection point” applicable across various post-stroke complications.

Our findings have immediate clinical applicability. The identified threshold (CAR = 0.18) provides an objective, quantitative criterion for triaging patients into risk categories upon admission. We propose a two-tier approach:

Low-risk (CAR < 0.18): These patients have OR = 1.22 for SAP—modest elevation above baseline. Standard pneumonia prevention protocols (early mobilization, semi-upright positioning, oral hygiene) are appropriate. Aggressive interventions may not be cost-effective in this group.

High-risk (CAR ≥ 0.18): These patients have OR = 2.03—substantially elevated risk. They warrant intensified surveillance: (1) Enhanced dysphagia screening within 6 h; (2) Strict NPO until formal swallowing assessment; (3) Meticulous oral care protocols (chlorhexidine rinses every 6–8 h); (4) Early mobilization and upright positioning (head-of-bed ≥30°); (5) Consideration of prophylactic antibiotics in select very-high-risk patients (e.g., CAR > 0.30, severe dysphagia), though this requires validation in randomized trials.

Implementation is straightforward: CAR can be auto-calculated by laboratory information systems whenever CRP and albumin are ordered, with automatic alerts for values ≥0.18. This requires no additional blood draws, equipment, or costs.

Importantly, CAR’s performance (AUC = 0.832) approaches that of comprehensive clinical scores while being objectively measurable and immediately available. The substantial improvement when added to A2DS2 (AUC: 0.764 → 0.859) demonstrates that a simple laboratory biomarker meaningfully enhances clinical assessment, making CAR particularly valuable in resource-limited settings or high-volume periods.

Strengths include: (1) Large, well-characterized cohort with comprehensive data; (2) Rigorous application of advanced non-parametric methods (GAM, two-piecewise regression) to identify threshold effects; (3) Systematic exclusion criteria minimizing confounding; (4) Independent adjudication of SAP diagnoses; (5) Comprehensive covariate adjustment; (6) Multiple sensitivity analyses confirming robustness.

Limitations must be acknowledged: (1) Single-center retrospective design limits generalizability—prospective multicenter validation is essential; (2) CAR was measured only once at hospital admission, which may not adequately capture the dynamic immunometabolic changes occurring during the biphasic post-stroke immune response. Both CRP and albumin are highly fluid biomarkers in the acute phase: CRP rises rapidly within 6–12 h of inflammatory stimulus and peaks at 48–72 h, while albumin declines progressively due to suppressed synthesis and increased vascular permeability. Consequently, a single admission measurement may either precede or lag behind the true “immunometabolic tipping point”—the temporal window of maximal vulnerability for SAP. Serial measurements at 48 and 72 h post-admission could potentially capture the trajectory of immunometabolic deterioration and provide superior predictive value. Serial laboratory data were not available in this retrospective cohort; future prospective studies should evaluate whether a rising CAR trajectory (rather than a single admission value) more accurately identifies patients entering the high-risk phase of stroke-induced immunodepression; (3) Despite rigorous adjustment, residual confounding by unmeasured factors cannot be excluded; (4) We used standard CRP rather than hs-CRP; (5) The threshold value (CAR ≈ 0.18) was derived and validated within the same dataset—it has not been confirmed in an independent external cohort, which limits our confidence in its generalizability and stability across different patient populations, healthcare settings, and geographic regions; external prospective validation is an essential prerequisite before clinical implementation; (6) The study evaluated only in-hospital SAP incidence without capturing long-term outcomes such as 30-day or 90-day mortality, long-term functional prognosis (e.g., modified Rankin Scale at 3 or 6 months), infection recurrence, or quality of life—consequently, the full prognostic value of CAR in the context of overall AIS outcomes remains to be established; (7) We did not evaluate whether CAR-guided interventions improve outcomes—only that CAR predicts risk.

While no statistically significant interactions were detected, a trend toward effect modification by atrial fibrillation was observed (*p* = 0.0658), with slightly lower OR in AF patients (1.38) compared to those without (1.69). This may reflect that in cardioembolic strokes, mechanical and hemodynamic factors driving SAP risk are more dominant, potentially diluting CAR’s relative contribution. Future studies with larger sample sizes should definitively assess this potential interaction.

Several research priorities emerge: (1) Prospective multicenter validation of the CAR = 0.18 threshold in diverse populations; (2) Randomized controlled trials testing CAR-guided preventive strategies; (3) Dynamic CAR monitoring studies; (4) Mechanistic investigations correlating CAR with direct immune function measures to validate the “immunometabolic vulnerability” hypothesis; (5) Combination biomarker panels; (6) Health economic analyses quantifying cost-effectiveness; (7) Investigation of whether interventions targeting CAR components (anti-inflammatory therapy, nutritional supplementation, albumin infusion) can modify risk; (8) Prospective studies incorporating long-term outcome measures—including 30-day and 90-day mortality, functional outcomes (modified Rankin Scale), infection recurrence, and quality of life—to comprehensively evaluate CAR’s role in overall AIS prognosis beyond in-hospital SAP incidence.

## 5 Conclusion

This study demonstrates that CAR exhibits a non-linear, threshold-dependent association with SAP at a clinically meaningful inflection point of 0.18. This threshold represents a critical “immunometabolic tipping point” where the confluence of inflammatory immunosuppression and metabolic depletion creates maximal vulnerability. Below this threshold, compensatory mechanisms remain relatively intact (OR = 1.22). Above it, the immune system enters a failure zone with dramatically escalated risk (OR = 2.03).

CAR provides superior predictive performance (AUC = 0.832) compared to individual markers and comparable to comprehensive clinical scores, while being immediately accessible from routine laboratory tests. The consistency across subgroups supports universal applicability without requiring subgroup-specific adjustments.

The identification of this threshold transforms CAR from an interesting association into a potentially practice-changing tool, providing clinicians with a specific, evidence-based criterion for risk stratification that can guide resource allocation and preventive intervention intensity. However, several important limitations must be acknowledged before clinical translation: the CAR threshold of 0.18 requires external validation in independent, multicenter cohorts to confirm its stability and generalizability, and future studies should incorporate long-term outcome measures—including mortality, functional recovery, and quality of life—to fully characterize CAR’s prognostic value in the broader context of AIS care. Future prospective studies validating this threshold and randomized trials testing CAR-guided interventions are essential next steps to establish whether this biomarker-driven approach can meaningfully reduce SAP burden and improve stroke outcomes.

## Data Availability

The raw data supporting the conclusions of this article will be made available by the authors, without undue reservation.
